# Early Biofilm Formation on UV Light Activated Nanoporous TiO_2_ Surfaces* In Vivo*

**DOI:** 10.1155/2018/7275617

**Published:** 2018-11-22

**Authors:** Nagat Areid, Eva Söderling, Johanna Tanner, Ilkka Kangasniemi, Timo O. Närhi

**Affiliations:** ^1^PhD Student, Department of Prosthetic Dentistry and Stomatognathic Physiology, Institute of Dentistry, University of Turku, Finland; ^2^Professor, Institute of Dentistry, University of Turku, Finland; ^3^Senior Lecturer, Department of Prosthetic Dentistry and Stomatognathic Physiology, Institute of Dentistry, University of Turku, Finland; ^4^Department of Oral and Maxillofacial Diseases, Turku University Hospital, Turku, Finland; ^5^Adjunct Professor, Turku Clinical Biomaterials Centre, University of Turku, Finland; ^6^Professor, Department of Prosthetic Dentistry and Stomatognathic Physiology, Institute of Dentistry, University of Turku, Finland; ^7^Chief Hospital Dentist, Department of Oral and Maxillofacial Diseases, Turku University Hospital, Turku, Finland

## Abstract

**Purpose:**

To explore early* S. mutans* biofilm formation on hydrothermally induced nanoporous TiO_2_ surfaces* in vivo* and to examine the effect of UV light activation on the biofilm development.

**Materials and Methods:**

Ti-6Al-4V titanium alloy discs (n = 40) were divided into four groups with different surface treatments: noncoated titanium alloy (NC); UV treated noncoated titanium alloy (UVNC); hydrothermally induced TiO_2_ coating (HT); and UV treated titanium alloy with hydrothermally induced TiO_2_ coating (UVHT).* In vivo* plaque formation was studied in 10 healthy, nonsmoking adult volunteers. Titanium discs were randomly distributed among the maxillary first and second molars. UV treatment was administered for 60 min immediately before attaching the discs in subjects' molars. Plaque samples were collected 24h after the attachment of the specimens. Mutans streptococci (MS), non-mutans streptococci, and total facultative bacteria were cultured, and colonies were counted.

**Results:**

The plaque samples of NC (NC + UVNC) surfaces showed over 2 times more often* S. mutans* when compared to TiO_2_ surfaces (HT + UVHT), with the number of colonized surfaces equal to 7 and 3, respectively.

**Conclusion:**

This* in vivo* study suggested that HT TiO_2_ surfaces, which we earlier showed to improve blood coagulation and encourage human gingival fibroblast attachment* in vitro*, do not enhance salivary microbial (mostly mutans streptococci) adhesion and initial biofilm formation when compared with noncoated titanium alloy. UV light treatment provided Ti-6Al-4V surfaces with antibacterial properties and showed a trend towards less biofilm formation when compared with non-UV treated titanium surfaces.

## 1. Introduction

Replacing missing teeth with dental implants is a common treatment modality with a predictable clinical outcome. A ninety-five percent survival rate after at least 10 years of follow-up has been reported [1]. However, implant associated infection is a common complication of dental implant treatment. There is increasing evidence that infections caused by oral bacteria are frequently a reason for implant failures [2, 3]. Peri-implantitis is an inflammatory disease that affects both soft tissues and supporting marginal bone around an implant, whereas peri-implant mucositis is a reversible inflammatory reaction restricted only to the peri-implant mucosa [4, 5]. This inflammatory phenomenon is a result of biofilm formation on the implant surface. Peri-implantitis and peri-implant mucositis have been reported in 18.8% and 63.4% of implant patients, respectively [6].

Biofilm formation is a process where microorganisms are irreversibly attached to a surface, interface or to each other, and produce extracellular polymers that facilitate attachment and matrix formation [7, 8]. The surface of transmucosal implant components are exposed to the oral environment containing saliva, gingival crevicular fluid, and microorganisms. Adherence of oral bacteria to solid surfaces is initiated by the adhesion of the early Gram-positive bacterial colonizers such as streptococci, which can further facilitate the binding of secondary bacterial colonizers leading to formation of an anaerobic Gram-negative microbial environment [9].* Streptococcus *spp. and* Actinomyces naeslundii* have been recognized as early colonizers on different implant material surfaces* in vivo *[10]. More mutans streptococci have been detected around infected implants compared to healthy implant sites [11].* S. mutans* is usually associated with caries occurrence, but it is also found in peri-implant biofilms due to its ability to produce an insoluble polymer matrix, survive at low pH values, and form biofilm with high affinity to solid surfaces such as implant components [12, 13].

Treatment of peri-implantitis by mechanical debridement of implant surfaces alone is difficult due to implants having various designs and surface textures. Along with the emergence of multiple antibiotic resistant strains of biofilm-associated microorganisms [14], routine antibiotic treatment is insufficient to eradicate implant associated infections. Prevention of peri-implant mucositis and peri-implantitis should be the primary goal in the management of these conditions. Sound adherence of gingival tissue to implant or abutment surfaces might prevent biofilm formation in peri-implant environments. Nanoporous TiO_2_ coatings have been found to enhance soft tissue attachment on titanium surfaces [15, 16]. Enhanced tissue adhesion is based on surface reactivity and proper nanotopography which together assist protein adsorption and cellular attachment [17–19]. Furthermore, antibacterial surface modification of implant biomaterials has gained more attention in the prevention of bacterial colonization and biofilm formation [20, 21]. Surface modifications of implant transmucosal components can inhibit bacterial biofilm adhesion [22], which subsequently improve soft tissue attachment [23, 24], and preserve alveolar ridge [25]. Among these various modifications, photocatalysis on TiO2 coating is considered a viable alternative approach to achieve antibacterial activity on biomaterials [26–28].

Photocatalysis of TiO_2_ coatings on solid surfaces provides self-cleaning, self-sterilizing capabilities, and antibacterial properties through its photoinduced hydrophilicity and decomposition reaction [29]. Photocatalytic TiO_2_ coatings have been demonstrated to be effective in biomedical applications due to their superhydrophilic and potential bactericidal properties, both induced by UV illumination [30]. When anatase TiO_2_ is irradiated with ultraviolet light (UV), electron-hole pairs are produced and reactive oxygen species (ROS) are generated. The hole in the valence band can react with water or hydroxide ions adsorbed on the surface to produce hydroxyl radicals (OH^−^) and the electron in the conduction band can reduce O_2_ to generate superoxide ions (O_2_^−^). These extremely reactive holes can act on the cell walls of nearby bacteria causing cell wall damage and eventually lead to cell death [31]. Several methods have been developed to obtain photocatalytically active TiO_2_ coatings [30, 32]. Among these, hydrothermal treatment (HT) has the advantage of being a relatively simple and flexible chemical coating method to produce anatase crystalline TiO_2_ coating [33]. We have previously shown that HT induced TiO_2_ surface promotes blood coagulation and human gingival fibroblast attachment. These responses were further improved after UV light activation [34, 35]. However, the same properties that enhance cellular adhesion may promote bacterial cell attachment and subsequent biofilm formation as well. In a previous laboratory experiment with sol-gel derived TiO_2_ surface treatment, this was however not the case [22]. On the other hand, UV light activation can introduce antimicrobial activity on HT induced TiO_2_ surfaces, which may inhibit biofilm formation. The purpose of the present study was to explore* in vivo* early* S. mutans* biofilm formation on HT induced nanoporous TiO_2_ surfaces and to examine the effect of UV light activation on the biofilm development. We chose* S. mutans* as the test organism since it is present in high counts in peri-implant biofilms. We have previously shown that while HT surface treatment introduces nanoporous TiO_2_ surface structure it also improves surface wettability, which was demonstrated by lower water contact angles. UV light treatment turns nanoporous TiO_2_ surfaces superhydrophilic [34, 35]. Our hypothesis is that these superhydrophilic surfaces hinder* S. mutans* adhesion and decrease biofilm formation when compared with non-UV treated HT induced TiO_2_ or commercially pure titanium surfaces. Furthermore, it is hypothesized that HT induced TiO_2_ surface* per se* does not enhance biofilm formation when compared with commercially pure titanium. 

## 2. Materials and Methods

### 2.1. Sample Preparation

**Figure 1:**
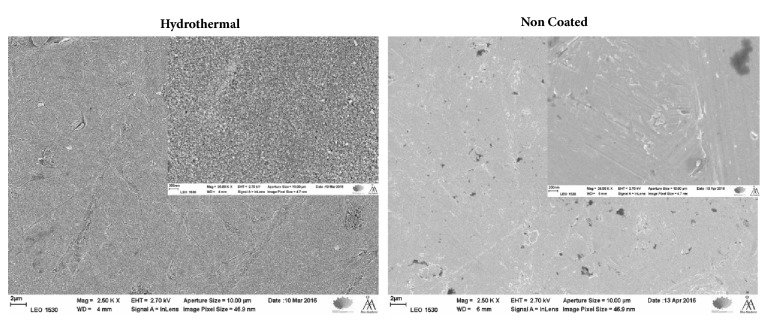
SEM images of hydrothermally induced TiO_2_ coatings and non-coated titanium alloy surfaces at low and high magnification.

Titanium (Ti-6Al-4V) alloy discs with a diameter of 4 mm and height of 1 mm were used in this study (n = 40). The discs were first ground using silicon carbide grinding paper of 1200 grit with an Ra value of 0.15*μ*m, ultrasonically washed with acetone for 5 min and then in ethanol for 5 min, and dried in air before any surface treatments were carried out. Two main groups, noncoated titanium alloy and nanoporous titanium dioxide surfaces (TiO_2_) obtained by the HT coating method as described earlier, were used as substrates [34, 35]. In brief, a hydrothermal suspension was prepared by dissolving titanium dioxide (TiO_2_), purified water, 1:10 diluted tetra methyl-ammonium hydroxide (TMAH) (N(CH_3_)_4_^+^ OH)^−^ and mixed for 5 min. Titanium discs were laid at the bottom of Teflon containers consisting of a Teflon inner vessel and a stainless-steel jacket; within this the hydrothermal suspension was added. Then, the vessel was kept at 150 ± 10°C in a constant-temperature oven for 48 h. Subsequent to the hydrothermal treatment period, the titanium discs were removed from the vessel and cooled in air. The discs were washed with distilled water in an ultrasonic bath for 10 min. Then the bottom surfaces of all discs were subjected to sandblasting with large grit aluminum oxide particles (250-500*μ*m) using an air abrasion machine (LM Pro power, Pargas, Finland) to enhance their attachment on the teeth surfaces. The sandblasting process of the samples was performed at distance of 3 mm for 20 s using 5 bars of air pressure at a 90° angle. In both groups (n=20) half of the substrates were treated with UV light for 60 min under ambient conditions using a 36 W puritec HNS germicidal ultraviolet lamp (Osram GmbH; Germany), with a dominant wavelength of 254 nm. Consequently four groups with different surface treatments (one control and three experimental discs) were obtained: noncoated titanium alloy (NC); UV treated noncoated titanium alloy (UVNC); hydrothermally induced TiO_2_ coating (HT); UV treated titanium alloy with hydrothermally induced TiO_2_ coating (UVHT). Scanning electron micrographs (SEM) were taken to examine the surface topography of the NC and HT substrate surfaces (Figure 1).

### 2.2. Subjects, Study Design, and Plaque Collection

**Figure 2:**
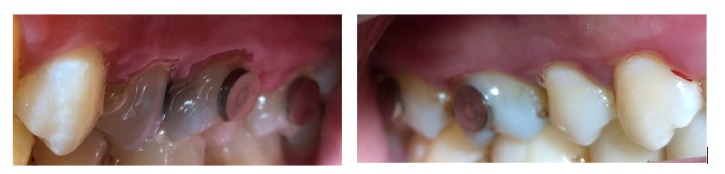
Titanium alloy discs attached to the buccal surfaces of maxillary molars.

*In vivo* plaque formation on four substrates with different surface treatments was studied in 10 healthy, nonsmoking adult volunteers (6 males, 4 females, mean age 39.7 years, ranging from 25 to 56 years). All the subjects were tested for* S. mutans* by collecting stimulated whole saliva for bacterial cultivation. The UV treatment was administered immediately (fresh surface) before attaching the discs in subjects' molars. Titanium discs were attached on subjects' buccal surfaces of their maxillary molars (Figure 2). The subjects were advised not to brush their teeth and not to use xylitol-containing products or antimicrobial mouthrinses during the plaque accumulation period. Each subject was advised to maintain their normal diet during the test period, but one day before and during the experiment, sucrose-containing cookies, chocolate, or candies could be consumed 3-5 times a day. This supposedly promoted adhesion of* S. mutans* to the materials [12]. None of the participants used antimicrobial drugs during the study.

The maxillary molars and premolars were professionally cleaned with pumice to remove plaque and pellicle. An area of the size of the specimen on the buccal surface of the tooth was etched with 37% orthophosphoric acid for 30 s, rinsed, and dried thoroughly. Bonding agent was applied (Scotch bond, 3M ESPE, Deutschland GmbH) and then light cured for 10 s. After that the titanium disc was attached on the conditioned tooth surface with light cured flowable composite resin for 20 s (Filtek™ Bulk Fill Flowable Restorative, 3M ESPE, Deutschland GmbH). Sharp edges were rounded using rotating polishing instruments and water cooling. The adherent plaque was collected according to a previously used method [36]. Briefly, after 24 h the outer (top) surface of attached titanium was gently rinsed with saline, and plaque was collected by rubbing the surface of each substrate with three applicator sticks (Quick-Stick® Dentsolv AB, Saltsjö-Boo, Sweden) containing approximately 4 *μ*l of NaCl solution. Care was taken not to touch the outer unpolished sides of the discs with the sticks. The tips of the sticks were cut off and collected into a tube containing 900 *μ*l of TSB. The samples were stored in −70°C before cultivation. One specimen was lost during plaque collection. A sample of stimulated saliva was collected using a paraffin wax chewing stimulation method for the assessment of salivary counts of* S. mutans*. Then, 100 *μ*l of the saliva was inoculated into 900 *μ*l of tryptic soy broth (TSB) and stored as frozen. After plaque collection the specimens were removed, and the excess composite was removed using hand instruments and rotating polishing instruments. Finally, fluoride varnish was applied on the polished enamel surfaces. All clinical procedures were performed by one investigator (NA).

All subjects (10) received all four materials (NC, UVNC, HT, and UVHT) and the surface area of the specimens was equal in all substrates. Titanium discs were randomly distributed among the maxillary first and second molars. Blinding was, however, applied to sample culturing and identification. The primary outcome measures were counts of* S. mutans*, and the secondary outcome measures were total of streptococci or “non-mutans streptococci”, which are important biofilm components in early oral biofilm.

### 2.3. Microbiological Analyses

The microbiological analyses were performed by a laboratory assistant and an experienced microbiologist. Both were blinded as to the sample coding. Cultivation procedure was initiated by thawing and vortexing the transport tubes of the plaque and saliva samples thoroughly. To detach the bacteria from the collection tips, the samples were treated in an ultrasonic bath for 10 s. Ten microlitre aliquots of serial tenfold dilutions of the plaque samples were plated on agar plates. MS were cultured on Mitis salivarius agar containing bacitracin (MSB, Becton, Dickinson and Company, Le Pont de Claix, France) [37].

The plates were incubated for 2 days in a 7% CO_2_ atmosphere at 37°C and* S. mutans* was identified on the basis of colony morphology and counted using a stereo microscope. The identification of* S. mutans *and* S. sobrinus* was performed as described earlier [38, 39]. Low counts of* S. sobrinus* was detected from the samples of only one subject. The counts were combined with* S. mutans* counts. Non-mutans streptococci were cultured for 2 days in air on Mitis salivarius agar at 37°C. All streptococcal-like colonies were counted as non-mutans streptococci. Total facultatives were cultured for 3 days anaerobically on blood agar (obtained from Turku University Hospital) at 37°C. All colonies were counted.

### 2.4. Statistical Analysis

Statistical analysis to compare the number of* S. mutans* CFU among the experimental groups was performed using the SPSS v.23.0 software package (IBM SPSS Inc.), by analyzing the differences among several means, the data were analyzed with one-way analysis of variance (ANOVA) followed by Tukey's* post hoc* test. Differences were considered significant at a 95% confidence level, with p-values below 0.05.

## 3. Results

Noncoated Ti-6Al-4V (NC) surfaces showed over 2 times more* S. mutans* in the early biofilm when compared with the hydrothermally (HT) induced nanoporous TiO_2_ surface. The numbers of colonized surfaces on NC and HT surfaces were equal to 7 and 3, respectively.

Figure 1 shows SEM images of the substrate surfaces. The NC substrates showed a smooth surface with some grinding lines spread out over the surfaces. The HT surfaces had a uniform smooth surface texture fully covered with the coating crystals consisting of nearly spherical nanoparticles of 20-50 nm. The surface did not change in appearance as a result of the UV treatment, and all the UV and NUV have the same surface morphology.

**Figure 3:**
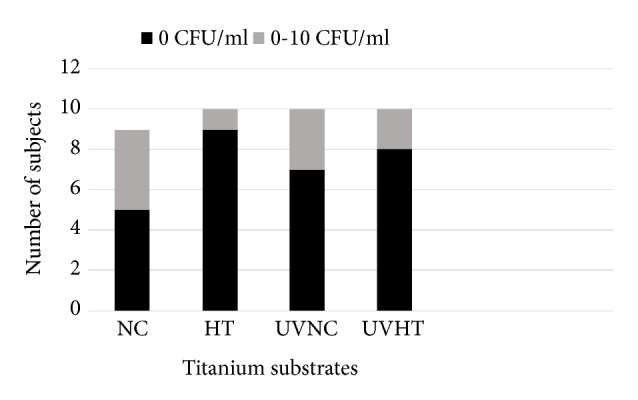
Number of subjects having no (0 CFU/ml) or low (< 10 CFU) MS counts in the plaque collected from the titanium alloy substrates: Non-coated titanium alloy (NC), hydrothermally induced TiO_2_ coating (HT), UV treated non-coated titanium alloy (UVNC) and UV treated titanium alloy with hydrothermally induced TiO_2_ coating (UVHT).

*S. mutans* was detected in the saliva of 7 out of 10 subjects. Three subjects showed no salivary* S. mutans* counts (0 colony-forming units (CFU)/ml), two showed low counts (<10^5^ CFU/ml), and five showed high counts (>10^5^ CFU/ml). All subjects with salivary* S. mutans* present showed some adherence of it to the studied materials. The distribution of subjects according to* S. mutans* counts found on the studied materials is illustrated in Figure 3. The mean log CFU counts (±SD) were 0.35± 0.4 for NC, 0.07± 0.2 for HT, 0.25 ± 0.4 for UVNC, and 0.16± 0.3 for UVHT (not statistically significant differences). However after cultivation, the plaque samples of noncoated groups (NC and UVNC) showed more often* S. mutans* in the biofilms than the coated hydrothermal groups (HT and UVHT) with the number of colonized surfaces equal to 7 and 3, respectively.

**Figure 4:**
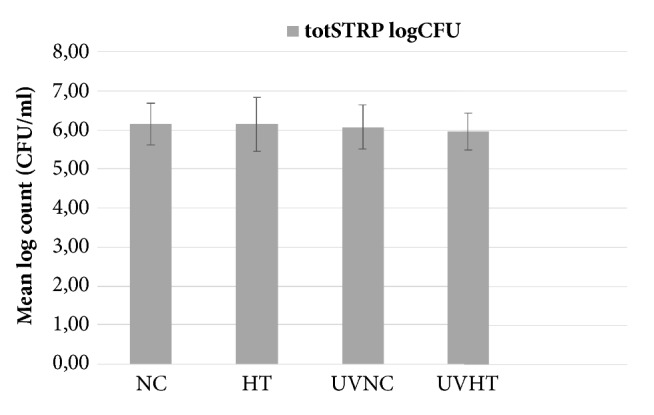
Mean logarithmic CFU counts (±SD) of non-mutans streptococci in the plaque collected from the studied materials: Non coated titanium alloy (NC), hydrothermally induced TiO_2_ coating (HT), UV treated non-coated titanium alloy (UVNC) and UV treated titanium alloy with hydrothermally induced TiO_2_ coating (UVHT).

**Figure 5:**
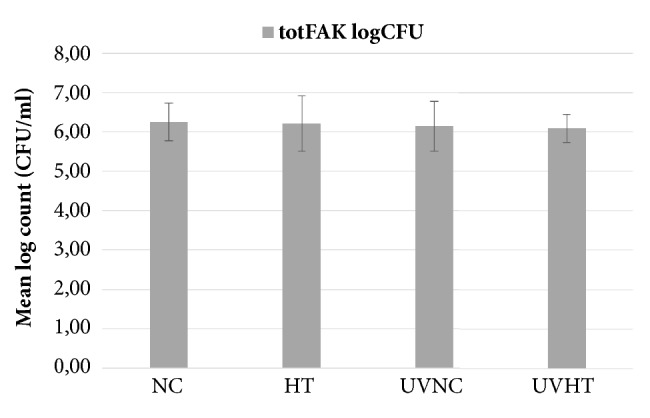
Mean logarithmic CFU counts (±SD) of total facultative bacteria in the plaque collected from the studied materials: Non coated titanium alloy (NC), hydrothermally induced TiO_2_ coating (HT), UV treated non-coated titanium alloy (UVNC) and UV treated titanium alloy with hydrothermally induced TiO_2_ coating (UVHT).

The results of the microbiological examinations of non-mutans streptococci and total facultative bacteria found from the samples of the studied materials are shown in Figures 4 and 5. No statistically significant differences were found between the groups. UVHT showed the lowest mean for both non-mutans streptococci and total facultative bacteria counts and NC showed the highest mean counts ((5.97 ± 0.5 and 6.09 ± 0.4) and (6.16 ± 0.5 and 6.26 ± 0.5), respectively). This trend was, however, not significant.

## 4. Discussion

In this study, the hydrothermally induced nanoporous TiO_2_ surfaces inhibited* S. mutans* adhesion and decreased biofilm formation when compared with noncoated titanium alloy.

The counts of non-mutans streptococci and total facultative bacteria were approximately similar on all studied substrates. In the 24 h plaque samples, the number of facultatives most likely reflects the number of streptococci. Previous studies have shown that healthy peri-implant sockets are mainly colonized by oral streptococci which constitutes from 45% to 86% of supra- and subgingival peri-implant sulcus microbiota.* Actinomyces *as well as* Neisseria* and* Rothia* species have also been frequently isolated [40, 41]. Diaz et al (2006) [42] have found that the initial colonizer on enamel surfaces are mainly streptococci followed by* Neisseria pharyngis* and* Gemella haemolysans*. These bacteria were considered by the authors to be a core group, providing the basis for the subsequent colonization of facultative and obligate anaerobes. Similarly, in the present study, streptococci appear to be the predominant species in a 24 h plaque on the studied substrates.

A microbial biofilm is considered an essential step in the initiation of peri-implant disease [43]. It can be affected by many factors, including local factors of implant and or abutment surface topography, as well as oral environment factors of saliva and protein [44]. The influence of surface properties such as surface roughness and surface chemistry on bacterial adhesion has been largely investigated [45, 46]. The surface roughness has been recognized as the predominant factor for biofilm formation on implant surfaces, as more biofilm is formed on rough modified surfaces compared with smooth surfaces [41, 45]. In our study the amount of plaque accumulation based on the counts of non-mutans streptococci and total facultative bacteria showed no difference among the experimental groups, which may be explained by surface roughness values of the studied substrates which ranged from 0.15 to 0.2 *μ*m. These findings are in agreement with a previous study suggesting that a roughness (R_a_) value of 0.2 *μ*m is a threshold limit below which surface roughness has no major effect on the biofilm formation or colonization [47]. Moreover, in our study the surface roughness of studied substrates was the same before and after UV treatment since the UV treatment did not alter the existing topography, roughness, or other morphologic features [48]. The surface characteristics of implants, such as surface free energy (SFE), and hydrophilicity have been shown to play crucial roles in bacterial adhesion and biofilm formation [49]. High SFE have been shown to attract more microorganisms than low SFE materials. On the contrary, an opposite result has also been reported [50]. A previous* in vivo *study by Tanner et al. [36], which compared the early plaque formation on different restorative materials, showed that polyethylene, the low SFE material, harboured more microorganisms than higher SFE dental ceramic and restorative composites. It was demonstrated that, in the oral environment, polyethylene fiber reinforced composite (FRC) promotes plaque accumulation and adhesion of* S. mutans* more than glass FRC, restorative composite, and dental ceramic [36]. Our results are in agreement with these findings. We have previously shown that while HT surface treatment introduces nanoporous TiO_2_ surface structure, it also improves surface wettability, which was demonstrated by lower water contact angles and higher SFE compared to the NC titanium alloy surface. This HT induced TiO_2_ surface also promotes blood coagulation and human gingival fibroblast attachment [34, 35]. The results of the microbiological examinations in the present study showed no statistically significant differences among the experimental groups. Our results seem to be in agreement with Rochford et al. [51] who showed that SFE can be improved on implant surfaces for better osseointegration without leading to more bacteria adhesion.

The nanoscale modification of the implant surface has been widely investigated. This surface modification can alter the surface chemistry and topography of an implant surface, which influences the initial cell response at the cell-material interface and improves bioactivity and bactericidal properties [22, 52]. In our study HT samples showed almost no* S. mutans* in the biofilms, whereas* S. mutans* was found on nearly half of NC samples after cultivation. The plaque samples of noncoated groups (NC, UVNC) harboured more frequently* S. mutans* in the biofilm than the coated hydrothermal groups (HT, UVHT) with the number of colonized surfaces being equal to 7 and 3, respectively. UV treated TiO_2_ surfaces have demonstrated superhydrophilicity, stain-proofing properties, and bactericidal properties [29, 53, 54]. Furthermore UV light treatment on various topographical titanium surfaces has been shown to reduce the biofilm formation of wound pathogens [55]. In our result, plaque recovered from the UVHT samples showed the lowest counts for both non-mutans streptococci and total facultative bacteria counts whereas NC samples showed the highest counts, although the differences were not significant and the real antimicrobial effect of UV treatment could not be confirmed in an oral environment. These findings, however, are in accordance with the* in vitro* study by Gallarado-Moreno et al. (2009) [56] who reported that UV treatment of Ti-6Al-4V inhibits bacterial attachment without compromising the osteoblast cell response to this alloy. This is probably related to the fact that UV treatment converts already hydrophilic nanostructured Ti alloy surfaces to superhydrophilic and cleans the contaminated hydrocarbons that accumulate on titanium surfaces [54].

The number of subjects (10) in the present study is relatively low. HT induced TiO_2_ surfaces and UV light treatment seem to reduce bacterial colonization, but much larger number of subjects will be needed for thorough statistical evaluation. On the other hand, the clinical importance should be seen in this number of subjects. The possibility of adding an* in situ *self-cleaning and antibacterial feature to HT induced TiO_2_ surfaces with UV light treatment could minimize implant infection related complications.

## 5. Conclusions

The results of this experimental* in vivo* study suggested that hydrothermally induced nanoporous TiO_2_ surface, which we earlier showed to improve blood coagulation and encourage human gingival fibroblast attachment* in vitro*, does not enhance salivary microbial (mostly mutans streptococci) adhesion and initial biofilm formation when compared with noncoated Ti-6Al-4V surfaces. UV light treatment provided Ti-6Al-4V surfaces with antibacterial properties and showed a trend towards less biofilm formation when compared with non-UV treated titanium surfaces.

## Data Availability

The raw data used to support the findings of this study are available from the corresponding author upon request.
